# Predictors of visual recovery in patients with macular edema secondary to central retinal vein occlusion after treatment with Conbercept

**DOI:** 10.1186/s12886-021-02174-0

**Published:** 2021-11-22

**Authors:** Qi Zhang, Yinfen Hou, Xiao Cao, Rongrong Zhang, Yinping Liu, Chenghua Wei, Changfan Wu, Lixin Mei, Pengfei Zhang

**Affiliations:** Department of Ophthalmology, The First Affiliated Hospital of Wannan Medical College (Yijishan Hospital of Wannan Medical College), Wuhu, 241001 China

**Keywords:** CRVO-ME, Conbercept, Best corrected visual acuity, predictive factor

## Abstract

**Background:**

The use of Spectral domain optical coherence tomography (SD-OCT) to evaluate the predictors of visual acuity-recovery in patients treated with conbercept for macular edema (ME) secondary to central retinal vein occlusion (CRVO) has rarely been seen. We collected 26 CRVO-ME patients with different OCT measures at 6 months follow-up to identify the factors that are most strongly correlated with the best-corrected visual acuity (BCVA) post-treatment in CRVO-ME patients treated with conbercept.

**Purpose:**

To evaluate the effectiveness of intravitreal conbercept injections for the treatment of CRVO-ME and to determine the major predictors of best-corrected visual acuity (BCVA) post-treatment.

**Methods:**

A retrospective study methodology was used*.* Twenty-six eyes from 26 patients with CRVO-ME were enrolled in the study. After an initial intravitreal injection of conbercept (0.5 mg/0.05 ml), monthly injections for up to 6 months were given following a 1 + PRN (pro re nata) regimen. Data collected at monthly intervals included measurements of the logMAR BCVA, central subfield thickness (CST), macular volume (MV), photoreceptor layer thickness (PLT), outer nuclear layer thickness (ONLT), and the disrupted ellipsoid zone (DEZ). The correlation between BCVA, before and after injections, and each of CST, MV, PLT, ONLT, DEZ was analyzed.

**Results:**

The logMAR BCVA in months 3 and 6 post-injection was significantly improved relative to the baseline. In this same period the CST, MV, PLT, ONLT and DEZ were also significantly improved relative to the baseline. There was a negative correlation between PLT and logMAR BCVA at months 3 and 6 after treatment (r = − 0.549, *P* < 0.001; r = − 0.087, *P* < 0.001).

**Conclusion:**

Intravitreal injection of conbercept is an effective treatment for CRVO-ME. With 6 months of follow-up, logMAR BCVA and CST, MV, PLT, ONLT, DEZ improved. PLT was negatively correlated with the visual function in CRVO-ME patients after conbercept treatment, which may be a predictor of vision recovery in patients with CRVO-ME.

## Background

Central retinal vein occlusion (CRVO) is one of the leading causes of vision loss among people aged 50 years or older. The incidence rate of CRVO in this group is approximately 1.36% [1]. Macular edema (ME) is the most common complication and a major cause of visual impairment in people with CRVO [2].

During CRVO formation, vascular endothelial growth factor (VEGF) levels increase resulting in an increase in the permeability of the retinal vessel walls. This causes the breakdown of the blood retinal barrier and ultimately leads to ME [3]. Many studies have shown that intravitreal anti-VEGF therapy is considered safe and effective for the treatment of CRVO-ME [4, 5]. Conbercept, an anti-VEGF medication, is a humanized, soluble, VEGF receptor (VEGFR) protein comprising extracellular domain-2 of VEGFR1, and extracellular domains-3 and-4 of VEGFR-2. Intravitreal injection of conbercept has been proven safe and effective for the treatment of CRVO-ME [6, 7]. However, clinically, repeated administration and follow-up visits are required, and some patients do not respond to treatment or have a poor visual outcome after repeated administration [8, 9]. Thus, it is very important to explore the predictive factors of a successful clinical outcome from conbercept therapy.

SD-OCT has been demonstrated to be an effective technique for analyzing macular edema through the evaluation of retinal structures. For example, using this technique, Xu et al. discovered that the ganglion cell layer and the inner plexiform layer are associated with visual gains in patients with diabetic macular edema [10]. Liu et al. discovered that external limiting membrane integrity is a prediction factor of visual function in CRVO-ME patients that have received ranibizumab injections [11]. However, SD-OCT assessment is still rarely to predict the visual acuity in CRVO-ME patients after conbercept therapy.

In this study, we investigated the retinal microstructure in patients with CRVO-ME after treatment with intravitreal conbercept during six-months follow-up period. We aim to identify the factors that are most strongly correlated with the best-corrected visual acuity (BCVA) post-treatment.

## Materials and methods

### Patients and inclusion criteria

This was a retrospective study. The patient group in this study consisted of 26 individuals diagnosed with CRVO-ME (26 eyes). All patients were examined and treated in the Department of Ophthalmology, the First Affiliated Hospital of Wannan Medical College (Yijishan Hospital of Wannan Medical College), China, from February 2016 and December 2019. Inclusion criteria were: (1) fundus fluorescein angiography diagnosis of CRVO; (2) OCT showed macular edema with macular thickness > 250 μm; (3) no prior treatment for CRVO-ME; (4) intraocular pressure within the normal range; (5) no other eye diseases present, including severe cataract, glaucoma, uveitis, fundus diseases, myopia more than 3.00 diopters (D); (6) no history of eye surgery; and (7) no other serious medical diseases. All patients had a detailed understanding of the treatment benefits and risks, and of the study’s medical ethics requirements. The study conformed to the tenets of the Declaration of Helsinki and was approved by the Ethical Review Committee of the First Affiliated Hospital of Wannan Medical College (Yijishan Hospital of Wannan Medical College), China (LLSC-2021-095). Written informed consent was obtained from each patient prior to participation in the study.

### Treatments and ophthalmic examination

All patients initially received one intravitreal injection of 0.5 mg/0.05 mL of conbercept (Chengdu Kang Hong Biotech Co, Ltd., Sichuan, China) and PRN (pro re nata) thereafter. All patients were evaluated monthly, including fluorescein angiography (FA) and optical coherent tomography (OCT) (Heidelberg Engineering, Heidelberg, Germany). The diagnostic criteria are according to previous studies [12]. The occlusion of the vein trunk and avascular zones in the retina are according to the performance of FA. If persistent or recurrent edema was detected, the 1 + PRN regimen was adopted. A persistent or recurrent edema was defined as a relapse of the cystoid space at the foveal center and an increase of the foveal thickness to 250 μm [13, 14].

**Fig. 1:**
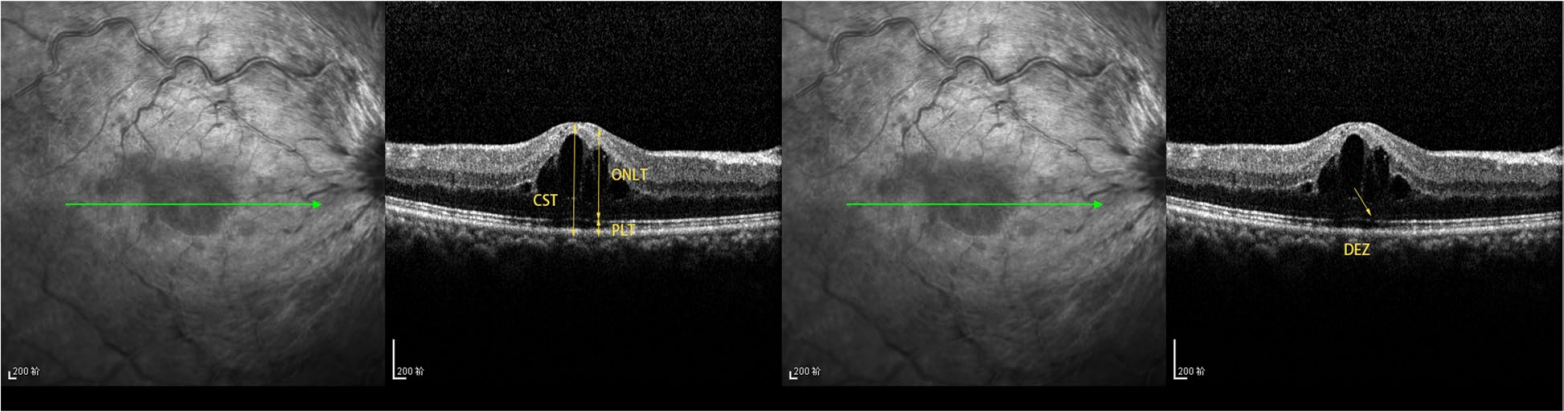
Spectral domain optical coherence tomography (SD-OCT) image of a CRVO-ME eye. SD-OCT image shows that central subfield thickness (CST), photoreceptor layer thickness (PLT), outer nuclear layer thickness (ONLT), and the disrupted ellipsoid zone (DEZ) (yellow arrow)

For all participants, we used OCT for data collection, including the logMAR BCVA, central subfield thickness (CST), macular volume (MV), photoreceptor layer thickness (PLT), outer nuclear layer thickness (ONLT), and disrupted ellipsoid zone (DEZ) **(**Fig 1**)**. Before the examination, the patients may be given Compound Topicamide eye drops for convenience of examination. During the examination, the examiner aims the lens at the patient's eye sitting directly in front of OCT, and lets the patient look at a fixed point of view and adjust the fixed point of view until a clear fundus image and OCT scanning line are presented on the fundus imaging display. At this time, relevant data required by OCT can be displayed and collected three times and took the average of them automatically according to the OCT instrument.

### Outcome measurements

All patients were followed for 6 months post-treatment. The logMAR BCVA, central subfield thickness (CST), macular volume (MV), photoreceptor layer thickness (PLT), outer nuclear layer thickness (ONLT), and disrupted ellipsoid zone (DEZ) were evaluated at the baseline, week 1, and at months 1, 3, and 6 post-treatment.

### Statistical analyses

All analyzes were conducted using SPSSv.18.0 for Windows (SPSS, Chicago, IL). Quantitative data that were normally distributed were analyzed using ANOVA. The correlation assesses between BCVA after medication and the OCT parameters using Pearson’s analysis. All statistical tests were two-sided. A *P*-value of < 0.05 was considered statistically significant.

## Results

### Demographic and clinical characteristics

Twenty-six eyes of 26 patients (11 females, 15 males) with CRVO-ME met the inclusion criteria between February 2016 and December 2019 (Table 1). The mean age of the participants was 57.70 ± 12.91 years. The mean intraocular pressure (IOP) of patients was 12.64 ± 2.13 mmHg. All patients received the 1 + PRN regimen. The total number of injections was 53, while the average number of injections per patient was 3.08 ± 0.21 (minimum = 1; maximum = 5). Three cases of subconjunctival hemorrhage occurred post-injection, with each undergoing self-absorption after 1 week of observation. There were no special complications in the remaining cases.

**Table 1:** Baseline characteristics of CRVO-ME patients

	CRVO-ME group
Numble	26
Age (years)	57.70 ± 12.91
Gender (F/M)	11/15
Race	Asian
Duration between initial IVC and baseline (weeks)	4.58 ± 0.31
Number of IVC	3.08 ± 0.21
Fluorescein angiography (ischemic/nonischemic)	9/17
BCVA (logMAR)	1.00 ± 0.73
IOP (mmHg)	12.64 ± 2.13
Mean CST (μm)	747.58 ± 149.38
Mean MV (μm^3^)	747.58 ± 149.38
Mean PLT (μm)	58.96 ± 8.56
Mean ONLT (μm)	432.58 ± 45.24
Mean DEZ (μm)	2921.04 ± 536.37

### Baseline and follow-up characteristics

**Fig. 2:**
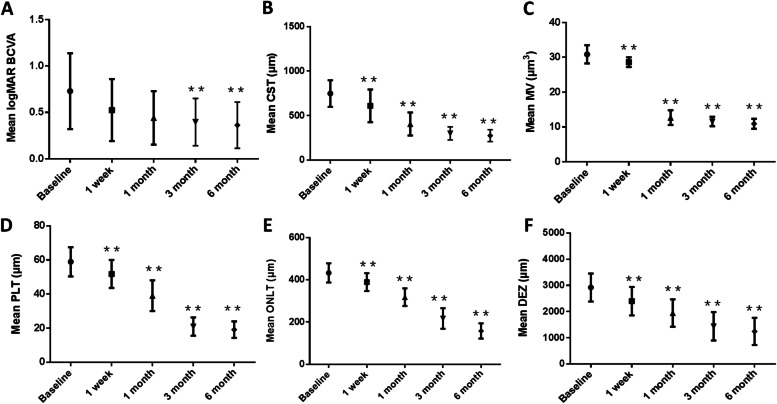
Comparison of BCVA (**A**), CST (**B**), MV (**C**), PLT (**D**), and ONLT (**E**) and DEZ (**F**) from baseline to month 6. The data are expressed as the mean ± SD, Compared with baseline (** *p* < 0.001)

The mean logMAR BCVA was 1.00 ± 0.73 at baseline, and the respective values for the mean logMAR BCVA in week 1, and months 1, 3, and 6 were 0.53 ± 0.33, 0.44 ± 0.29, 0.40 ± 0.25, and 0.36 ± 0.08. Comparison of the logMAR BCVA pre-injection and post-injection showed a statistically significant difference in months 3 (*P* = 0.007) and 6 (*P* = 0.003) post-injection (Fig. 2A).

The mean CST was 747.58 ± 149.38 μm at baseline, and the respective values for the mean CST in week 1, and in months 1, 3, and 6 were 609.92 ± 187.73 μm, 404.50 ± 129.49 μm, 299.50 ± 74.24 μm, and 274.08 ± 29.30 μm. Mean CST decreased between the baseline period and week 1 (*P* = 0.003), month 1 *(p ≤* 0.001), month 3 (*p ≤* 0.001), and month 6 (*p ≤* 0.001*)* post-injection. Mean CST decreased between week 1 post-injection and month 1 (*p ≤* 0.001), month 3 (*p ≤* 0.001), and month 6 (*p ≤* 0.001) post-injection. Mean CST also decreased between month 1 post-injection and both month 3 (*P* = 0.018) and month 6 (*P* = 0.004) post-injection (Fig. 2B).

The mean MV was 30.87 ± 2.63 μm^3^ at baseline, and the respective values for the mean MV in week 1, and in months 1, 3, and 6 were 28.62 ± 1.41 μm^3^, 12.70 ± 2.10 μm^3^, 11.57 ± 1.35 μm^3^, and 10.93 ± 0.52 μm^3^. Mean MV in decreased between the baseline and week 1 (*P* = 0.005), month 1 (*p ≤ 0.001*), 3 (*p ≤ 0.001*), and 6 (*p ≤ 0.001*) post-injection. Mean MV decreased between week 1 post-injection and month 1 (*p ≤ 0.001*), month 3 (*p ≤ 0.001*), and month 6 (*p ≤ 0.001*) post-injection. Mean MV also decreased between month 1 post-injection and month 6 post-injection (*P* = 0.023, Fig. 2C).

The mean PLT was 58.96 ± 8.56 μm at baseline, and the respective values for the mean MV in week 1, and in months 1, 3, and 6 were 51.85 ± 8.22 μm, 39.04 ± 9.03 μm, 20.92 ± 5.36 μm, and 19.15 ± 1.68 μm. PLT decreased between the baseline period and week 1 (*P* = 0.006), month 1 (*p ≤ 0.001*), month 3 (*P* = 0.006), and month 6 (*P* = 0.006) post-injection. PLT decreased between week 1 post-injection and month 1 (*p ≤ 0.001*), month 3 (*p ≤ 0.001*), and month 6 (*p ≤ 0.001*) post-injection. PLT also decreased between month 1 post-injection and both month 3 (*p ≤ 0.001*) and month 6 (*p ≤ 0.001*) post-injection (Fig. 2D).

The mean ONLT was 432.58 ± 45.24 μm at baseline, and the respective values for the mean MV in week 1, and in months 1, 3, and 6 were 389.31 ± 42.45 μm, 317.96 ± 41.57 μm, 217.04 ± 48.86 μm, and 157.92 ± 8.87 μm. The ONLT decreased between the baseline period and week 1 (*P* = 0.002), month 1 (*P ≤ 0.001*), month 3 (*P ≤ 0.001*), and month 6 (*p ≤ 0.001*) post-injection. The ONLT decreased between week 1 post-injection and month 1 (*P ≤ 0.001*), month 3 (*P ≤ 0.001*), and month 6 (*P ≤ 0.001*) post-injection. The ONL also decreased between month 1 post-injection and both month 3 (*P ≤ 0.001*) and month 6 (*P ≤ 0.001*) post-injection. Furthermore, the ONLT decreased between month 3 and month 6 post-injection (*P* = 0.001, Fig. 2E).

The mean DEZ is 2921.04 ± 536.37 μm at baseline, and the respective values for the mean MV in week 1, and in months 1, 3, and 6 were 2401.23 ± 543.33 μm, 1944.73 ± 522.30 μm, 1440.88 ± 542.88 μm, and 1245.42 ± 105.19 μm. The DEZ decreased between the baseline and week 1 (*P* = 0.002), month 1 (*P ≤ 0.001*), month 3 (*P ≤ 0.001*), and month 6 (*P ≤ 0.001*) post-injection. The DEZ decreased between week 1 post-injection and month 1 (*P* = 0.005), month 3 (*P ≤ 0.001*), and month 6 (*P ≤ 0.001*) post-injection. The DEZ also decreased between month 1 post-injection and both month 3 (*P* = 0.002) and month 6 (*P ≤ 0.001*) post-injection (Fig. 2F).

### Correlation analysis of BCVA after injection

**Fig. 3:**
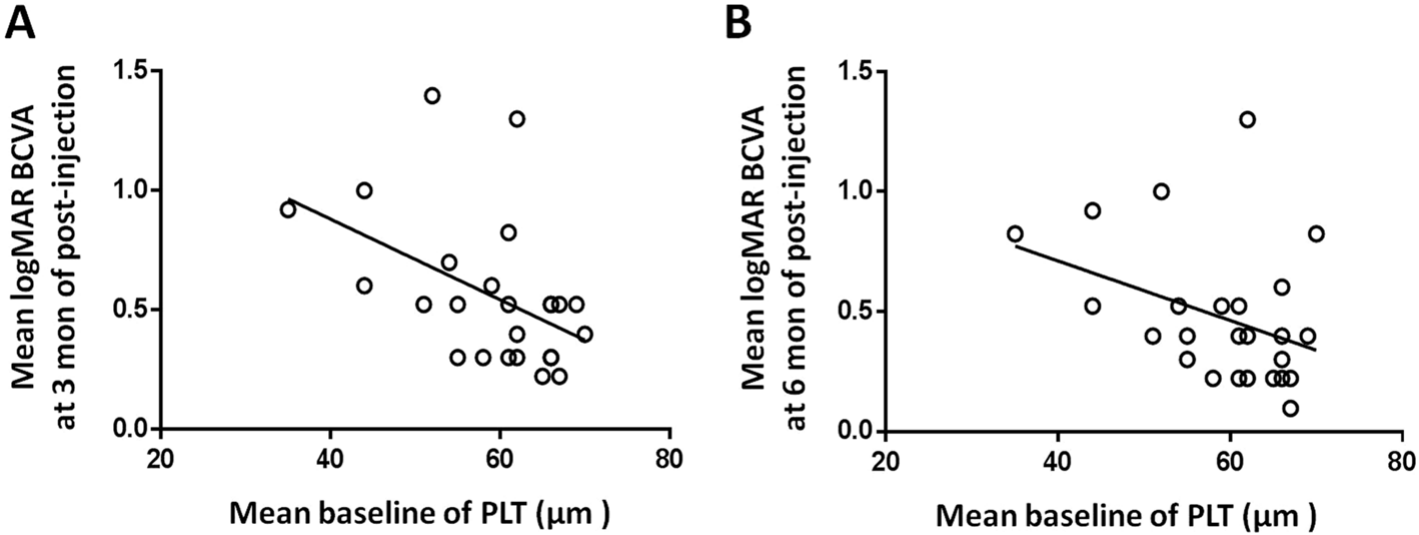
Association between baseline PLT and logMAR BCVA at month 3 of post-injection (r = − 0.549, *P* ≤ 0.001, Fig. A), Association between baseline PLT and logMAR BCVA at month 3 of post-injection (r = − 0.087, *P* ≤ 0.001, Fig. B)

The results of the Pearson’s correlation analysis showed that there was a statistically significant correlation between the baseline of PLT and the post-injection BCVA correlated at month 3 (*r* = − 0.549, *P* < 0.001) and month 6 (*r* = − 0.087, *P* < 0.001, Fig. 3).

## Discussion

Macular edema is the major complication secondary to central retinal vein occlusion that can lead to severe impairment of central vision [15]. Previous studies have shown that CRVO-ME can destroy the normal structure of macular causing photoreceptor dysfunction [16, 17]. Therefore, it is necessary to assess the changes in macular structure and visual function associated with gains in visual acuity after an intravitreal conbercept injection. In this study, we found that after conbercept injection, CST, MV, PLT, ONL, and DEZ all improved with observation time relative to baseline measurements. The mean logMAR BCVA improved significantly in the 3 months after treatment and stabilized by 6 months. Furthermore, we showed that the baseline of PLT and BCVA at months 3 and 6 after treatment have a negative correlation, which indicated that PLT may be a predictor for vision recovery of CRVO-ME.

Although treatment of macular edema typically follows a 3 + PRN regimen due to regional economic influences [18], our treatment regimen was monthly intravitreal conbercept injections for 1 month followed by 1 + PRN regimen. It was found that the structure and function of the macular area were improved relative to the baseline and the visual acuity improved over the duration of the study. This suggests that the treatment program is effective for treating CRVO-ME. The results reported here are consistent with previous studies [19, 20]. However, the macular edema has not yet disappeared significantly in many cases 1 week after the first injection in this study. The mean CST of greater than 400 μm 1 month after the first injection, less than 400 μm after 3 months after treatment. The results suggests that macular edema may not soon disappeared after treatment initiation in many cases. Previous studies discovered that CST does not soon decreased in RVO patients with the longer course, with the phenomenon of antagonism of VEGF or recurrent edema occurs [21]. Thus, it may be due to the longer course of RVO-ME, as well as ischemic cases in some patients, the macular edema has not yet disappeared in some cases after treatment.

Previous studies have noted that vision recovery was limited even though retinal thickness was reduced to normal levels after treatment [22, 23]. The reason for this is not clear. It is known that macular edema was noted mostly in the outer nuclear layer, causing photoreceptor cell loss and fovea dysfunction [23]. Akagi-Kurashige et al. found that macular photoreceptor abnormalities of RVO patients could cause a decrease in parafoveal cone density and disrupt the cone mosaic spatial arrangement [24]. Therefore, the assessment of the retinal layers, especially those consisting of photoreceptor layers, is useful for evaluating visual prognosis.

In our study, we found a negative correlation between BCVA and PLT. The photoreceptor layer is the area where photoreceptor cells are distributed on the OCT. Therefore, the variation of photoreceptor thickness reflects the loss of photoreceptors. Histologic studies have showed that macular edema secondary to RVO is mostly detected in the outer nuclear layer, along with cystoid spaces and causes liquefaction necrosis, which results in loss of photoreceptor cells and photoreceptor dysfunction in the fovea [25]. To recover visual acuity that was adversely affected by macular edema, a reduction in retinal thickness may be necessary. However, the loss of photoreceptor cells may lead to limited visual recovery. Therefore, preservation of the photoreceptor cells would thus be necessary [26].

In this study, we measured the thickness of the photoreceptor layer, and found there was a negative correlation between photoreceptor layer thickness and logMAR BCVA at months 3 and 6 after treatment. We hypothesized that macular edema secondary to CRVO may result in structural damages, disorganization, and loss of photoreceptor cells. Especially when macular edema exceeds its elastic limit of outer nuclear layer, the damage of photoreceptor cells is irreversible. Following macular edema subsiding, the thinning of the PLT may reflect the loss of photoreceptor cells, which was responsible for the limited improvement in vision. Of course, the sample size of this study is small and the observation time is short, which may lead to the deviation of the results. In the future, large samples, multi-center, long-term observations are needed to be to be carried out.

There are some methodological limitations that should be considered when evaluating the results of this study. First, the study was performed at a single center and the sample size was relatively small. A larger sample size and longer follow-up period will be needed to validate our results. Second, CRVO is a chronic disease, while the observation time in this study is short. It is therefore difficult to fully assess the long-term prognosis with conbercept treatment. Third, we manually measured the various retinal layer thicknesses, although automated software would have allowed a more objective evaluation and erased any potential bias.

In sum, the study demonstrated the efficacy of conbercept treatment for CRVO-ME using a 1 + PRN regimen. Improvements with observation time over the 6 months follow-up period were found in the central subfield thickness, macular volume, photoreceptor layer thickness, outer nuclear layer and disrupted ellipsoid zone. The mean logMAR BCVA are improved significantly in the 3 months after treatment and stabilized by 6 months post-treatment. PLT is associated with the visual function in CRVO-ME patients after conbercept treatment and may be a predictor for vision recovery.

## Data Availability

The data are available from the corresponding author upon reasonable request.

## Abbreviations

SD-OCT: Spectral domain optical coherence tomography
BCVA: Best-corrected visual acuity
CRVO-ME: Macular edema secondary to central retinal vein occlusion
CST: Central subfield thickness
MV: Macular volume
PLT: Photoreceptor layer thickness
ONLT: Outer nuclear layer thickness
DEZ: The disrupted ellipsoid zone
